# Chemical and Structural Analysis of Newly Prepared Co-W-Al Alloy by Aluminothermic Reaction

**DOI:** 10.3390/ma15020658

**Published:** 2022-01-16

**Authors:** Štefan Michna, Anna Knaislová, Iryna Hren, Jan Novotný, Lenka Michnová, Jaroslava Svobodová

**Affiliations:** Faculty of Mechanical Engineering, J. E. Purkyne University in Usti nad Labem, Pasteurova 3334/7, 400 01 Usti nad Labem, Czech Republic; stefan.michna@ujep.cz (Š.M.); iryna.hren@ujep.cz (I.H.); jan.novotny@ujep.cz (J.N.); lenka.michnova@ujep.cz (L.M.); jaroslava.svobodova@ujep.cz (J.S.)

**Keywords:** aluminothermic reaction, Co-W-Al alloy, carbides, structural analysis, chemical composition, scanning electron microscopy, EDS analysis

## Abstract

This article is devoted to the characterization of a new Co-W-Al alloy prepared by an aluminothermic reaction. This alloy is used for the subsequent preparation of a special composite nanopowder and for the surface coating of aluminum, magnesium, or iron alloys. Due to the very high temperature (2000 °C–3000 °C) required for the reaction, thermite was added to the mixture. Pulverized coal was also added in order to obtain the appropriate metal carbides (Co, W, Ti), which increase hardness, resistance to abrasion, and the corrosion of the coating and have good high temperature properties. The phase composition of the alloy prepared by the aluminothermic reaction showed mainly cobalt, tungsten, and aluminum, as well as small amounts of iron, titanium, and calcium. No carbon was identified using this method. The microstructure of this alloy is characterized by a cobalt matrix with smaller regular and irregular carbide particles doped by aluminum.

## 1. Introduction

### 1.1. Aluminothermic Reaction

The preparation of particular multicomponent alloys containing metals with melting points of 660 °C (aluminum) as well as high-melting point-metals such as cobalt (1495 °C), titanium (1668 °C), molybdenum (2623 °C), and tungsten (3422 °C) is very difficult [1,2,3,4]. Another problem with the preparation of these alloys containing aluminum-refractory metals is the extensive dispersion of density and the fact that these alloys do not form solid solutions with one another in most cases. One of the possibilities for the preparation of these multicomponent melts is an aluminothermic reaction.

An aluminothermic reaction is a highly self-propagating exothermic reaction that uses aluminum powder as the reducing agent [5]. The reaction takes place very quickly. In this process, the high affinity of aluminum for oxygen is used, and a more stable metallic oxide (Al_2_O_3_) is formed [6,7]. The general equation is:

Al + XO → Al_2_O_3_ + X + Q (1)

where Al is aluminum, XO is metal or non-metal oxide, Al_2_O_3_ is aluminum oxide, X is metal or non-metal, and Q is energy (heat generated by the reaction). Any oxide with a Gibbs free energy of formation higher than that of Al_2_O_3_ can be used as the reactant oxide (XO) [7,8].

This reaction can take place in environments without oxygen or under water, due to the zero-oxygen balance of the reaction [6,9]. In refractory metals such as chromium, titanium, or tungsten, it is necessary to dope the process thermally, for which thermite can be used. Thermite is a pyrotechnic mixture that burns at high temperatures. The most common composition of termites is pyroaluminum (ca. 25 wt.%) and iron (II,III) oxide (ca. 75 wt.%). By using this mixture, temperatures of up to 2500 °C can be achieved [10]. The aluminothermic reactions of various metal oxides (such as TiO_2_ [11,12,13,14], SiO_2_ [15,16,17,18], ZnO [19,20,21,22,23,24], Cr_2_O_3_ [25,26,27], CuO [28,29,30]) have been studied for many years. The aluminothermic reaction is useful in the production of metals and alloys, such as refractory ceramics, composite materials, nanowires and nanocoatings, ceramic coatings for metallic pipes, and railway welding [7,29,31,32].

A specific process in the aluminothermic production of alloys is the recovery of part of the refractory metals (W, Co, Ti) in the form of metal carbides [33,34,35,36]. In this case, the aluminothermic reaction is used specifically, where the charge added to the carbon crucible consists of individual powder components (WO_3_, Co_2_O_3_, coal, aluminum, etc.) according to the desired final composition. The mixture is heated in an electric furnace at 900 °C in a stream of argon, then the synthesis is completed by short rapid heating at 1000 °C (ignition for an aluminothermic reaction). After cooling, the obtained material is ground and sieved to the required granulometry of about 1 µm [33].

In this article, Co-W-Al alloy was prepared by the aluminothermic reaction. One of the disadvantages of the aluminothermic reaction, where metals are obtained from metal oxides, is the low purity of the final product (produced alloys). In the case of the Co-W-Al alloy, it can be assumed that the alloy, apart from the elements Co, W (partly in the form of carbides), Al, and C, will also contain a percentage of substances such as titanium, iron, and calcium (from CaF_2_).

### 1.2. Theoretical Aspects of the Structural Composition of the Co-W-Al System

In this section, the theoretical aspects of the structural composition of the Co-W-Al system and the effect of alloying elements on the structure and substructure of cobalt alloys will be discussed. From the point of view of evaluating the effect of alloying elements on the properties of cobalt alloys, it is necessary to state that cobalt has two allotropic modifications. Below 417 °C, it is hcp modification and above 417 °C it is fcc modification (K12—phase γ) [37]. Elements such as C, Ta, Nb, Zr, Ti, V, Fe, Mn, and Ni stabilize the structure of the γ phase with the K12 lattice even at temperatures below 417 °C [38,39].

The Al-Co binary diagram shows a solid solution of Al (fcc); a solid solution of α-Co (fcc); a solid solution of ε-Co (stable below 422 °C); and various binary phases: Al_9_Co_2_, Al_13_Co_4_, Al_3_Co, and Al_5_Co_2_ (formed by peritectic transformation from melt at 660 °C, 970 °C, 1090 °C, and 1180 °C, respectively). The diagram also shows the binary phase AlCo, which is formed congruently from the melt at approximately 50% of the cobalt content in the alloy [40]. The intermetallic phase Al_9_Co_2_ predominates at a high aluminum content, and the β-CoAl phase predominates at a decreasing aluminum content [41].

The Co-W binary diagram (calculated by Gui and Ost) shows a solid solution of W, a solid solution of α-Co (fcc), and a solid solution of ε-Co. α-Co and ε-Co are allotropic modifications of cobalt with the transformation temperature at 422 °C. The diagram shows two intermetallic phases: Co_3_W (hexagonal crystal structure) and Co_7_W_6_ (rhombohedral phase). The first mentioned phase, Co_3_W, is formed by the peritectoid reaction from the melt at 1093 °C. The second one, Co_7_W_6_, is more stable with a higher tungsten content [42,43].

Part of the ternary diagram of Co-Al-W at 900 °C shows the existence of a γ′ phase at 900 °C. In 2006, it was found that there is a Co_3_ (Al, W) phase in the Co-Al-W system. This phase was marked as γ′ and can coexist with the γ phase of the cobalt matrix; the difference in the lattice parameters between them is ≈0.53% [44,45]. As a result, the γ′ phase precipitates are coherent with the γ phase existing in Co alloys above 417 °C up to the melting point. These coherent precipitates of the γ′ phase increase the strength properties, which are maintained up to the melting point of cobalt. Ti and Ta have a strong stabilizing effect on the existence of the γ′ phase. This finding has led to the development of new Co superalloys with high-temperature applications, especially for aircraft engine turbine blades and discs. The γ′ phase precipitates are very fine (≈20 nm) and have a cuboidal character [45].

In the ternary system Co-Al-W, the intermetallic phases Co_3_W and Co_7_W_6_, referred to as μ phases, are present [46]. The presence of other alloying elements in the Co-Al-W ternary system, such as Mo, Nb, and Ta, promotes the precipitation of the μ phase at 1300 °C [46]. In the work of the authors [47], it was found that the presence of Cr in Co-30Ni-10Al-5Mo-2Ta alloys or Co-30Ni-10-Al-5Mo-2Ta-2Ti causes γ′ precipitates to have both cubic and globular character [48,49].

It has been found that the presence of Al significantly increases the oxidation resistance of Co-Al-W-based alloys [50,51]. Oxidation at 800 °C results in a typical layer in which the upper part is made up of Al_2_O_3_. According to the experimental results of the work [45], 13 wt.% Cr increases the oxidation resistance of Co-Al-W superalloys more than 40 times.

The presence of carbon in the aluminothermic reaction of Co-W-Al alloys aims to obtain carbides of the respective metals. For wear-resistant alloys in the Co-Cr-W-C system, carbide phases were identified at various temperatures more than 70 years ago, such as MC, M_2_C, M_7_C_3_, M_23_C_6_, M_6_C, M_12_C, and M_25_C, where M is the metal [52,53].

The existence, formation, and character of these carbide particles depend on temperature and time. The structure of cobalt superalloys is, in principle, formed by coherent precipitates of γ′ and μ phases. Due to the coherence of lattices with a high-temperature (Co matrix) γ phase, their aim is to ensure the preservation of strength properties up to the melting temperature of cobalt. The carbide phases present—in particular the M_26_C_6_ phase precipitated along the grain boundaries—are intended to ensure high creep properties [53].

## 2. Materials and Methods

In this work, a special multicomponent alloy Co70W12Al15 (composition in wt.%) was prepared using the aluminothermic reaction. Pure aluminum (coarse powder), tungsten oxide (WO_3_), cobalt dioxide (Co_2_O_3_), pulverized coal, and a slag-forming additive in the form of calcium fluoride (CaF_2_) were used as starting materials. 

The overall metallurgical process for the preparation of Co70W12Al15 alloy in the presence of carbon can be expressed by the following chemical reactions:

WO_3_ + 2Al → Al_2_O_3_ + W (2)

Co_2_O_3_ + 2Al → Al_2_O_3_ + 2Co (3)

3TiO_2_ + 4Al → 2Al_2_O_3_ + 3Ti (4)

The following reaction is also possible:

Fe_2_O_3_ + 2Al → Al_2_O_3_ + 2Fe (5)

Due to the presence of carbon, reactions also occur in part:

2W + 3C → W_2_C_3_ (6)

Ti + C → TiC(7)

3Co + 2C → Co_3_C_2_ (8)

Due to the presence of the elements Co, W, and Ti, complex carbides form in a certain stoichiometric ratio, such as:

W + Co + Ti + C→ (Co, W, Ti)C(9)

An X-ray fluorescence method was used to analyze the chemical composition of the produced Co70W12Al15 alloy; this was used to determine the chemical composition of the material. The measurement was performed using a portable hand-held X-ray spectrometer with a metal analyzer DELTA PROFESSIONAL SDD (Silicon Drift Det) (BAS Rudice, Blansko, Czech Republic). The DELTA PROFESSIONAL model uses compact X-rays with a power of 4W, an optimized anode, and the possibility of a maximum current of up to 200 µA.

The phase composition of Co70W12Al15 alloy was measured by X-ray diffraction analysis using a diffractometer PANalyticalX’Pert Pro (PANalytical, Almelo, The Netherlands), followed by evaluation in the X’PertHighScore 3.0 software package (PANalytical, Almelo, The Netherlands) using the PDF-2 2018 database. 

The metallographic cut was prepared for observing the microstructure. The sample was cut; ground by P80 to P2500 grinding papers (Hermes Schleifmittel GmbH, Hamburg, Germany); and polished by diamond pastes D3, D1, and D0.7. The sample was etched by a mixture of 10 mL HNO_3_ + 20 mL HCl + 30 mL H_2_O (it was prepared in our laboratory). The microstructure of the alloy was investigated using a LEXT OLS 3000 laser confocal microscope (Olympus, Sindzuku, Japan) and a scanning electron microscope Tescan Vega 3XMU (Tescan, Brno, Czech Republic) with an Oxford Instruments X-max 20 mm^2^ EDS analyzer (Oxford Instruments, High Wycombe, UK). To evaluate the mechanical properties, the samples were subjected to Vickers hardness measurements HV 0.1 (Vickers hardness with a load of 100 g) on a microhardness tester Shimadzu HMV-2 (Shimadzu, Kyoto, Japan).

## 3. Results

### 3.1. Analysis of the Chemical Composition of the Produced Alloy Co70W12Al15

The results of the analysis of the chemical composition of the Co70W12Al15 alloy are shown in Table 1. Three measurements were performed. X-ray fluorescence cannot determine the carbon content; instead, EDS analysis was performed on a scanning electron microscope to determine its amount. From Table 1, it can be stated that the alloy Co70W12Al15 is formed by the basic elements of the said alloy, namely: Co 68.06 ± 1.73%, Al 15.15 ± 0.27%, and W 12.62 ± 1.20%. Furthermore, due to the raw materials used and the alloy preparation technology used, Ca 2.71 ± 0.94%, Ti 2.08 ± 0.69%, and Fe 2.70 ± 0.38% were present.

Area EDS analysis identified the amount of carbon—approximately 6%. The content of elements determined by EDS analysis does not differ significantly from the content of elements determined by XRF.

From the results of the X-ray fluorescence method and EDS analysis on a scanning electron microscope, it can be stated that the alloy Co70W12Al15 consists of the basic elements of the alloy, namely: cobalt (approx. 68%), aluminum (approx. 15.5%), and tungsten (approx. 13%). In terms of the raw materials used and/or the alloy preparation technology used, mainly carbon (approx. 6.0%), calcium (approx. 2.7%), titanium (approx. 2%), and iron (approx. 2.7%) are present in the alloy. A small amount of residual oxygen (approx. 1.8%) is also identified in the alloy, which comes mostly from the aluminothermic reaction, both from the incomplete reduction of metals and from the rest of the completely unremoved slag in the form of alumina. It is thus a multicomponent alloy, where, in addition to the base metal cobalt and the alloying metals tungsten and aluminum, there are other impurity elements (Ti, Ca, Fe, C, O) present in units of percent.

**Figure 1:**
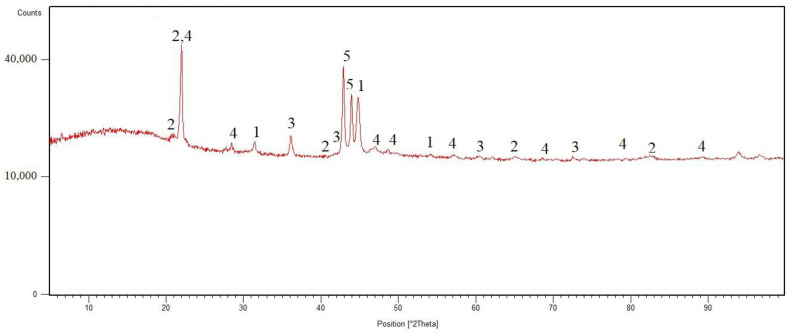
XRD pattern of Co70W12Al15 alloy: 1—AlCo; 2—WC; 3—TiWC_2_; 4—SiO_2_; 5—C.

The phase composition was measured by X-ray diffraction. The XRD pattern is shown in Figure 1. The Co70W12Al15 alloy consists of pure AlCo phase, tungsten carbide, titanium tungsten carbide, silica, and carbon.

### 3.2. Microstructure of the Produced Alloy Co70W12Al15

**Figure 2:**
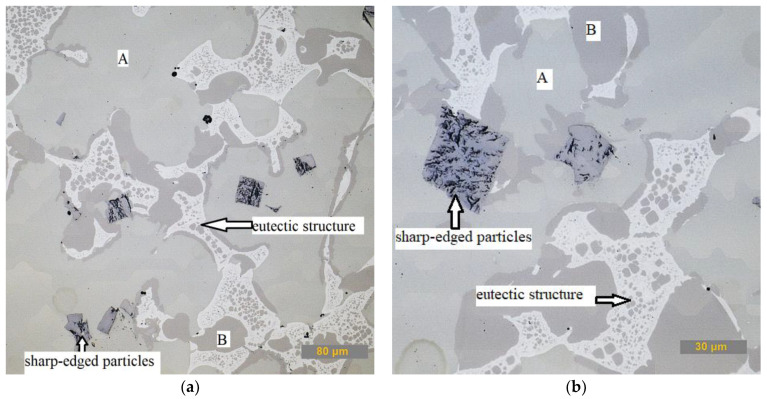
Microstructure of Co70W12Al15 alloy: (**a**) the overall microstructure; (**b**) detail of the microstructure with the occurrence of sharp-edged particles.

The microstructure of the Co70W12Al15 alloy was investigated on a metallographic cut using an OLS 3000 laser confocal microscope. The microstructure of the Co70W12Al15 alloy is characterized by the presence of two types of solid solutions. The first solid solution (labeled A in Figure 2a) is light in color with an inhomogeneous composition and darker fine particles. The second solid solution is darker with a homogeneous composition (labeled B in Figure 2a). Furthermore, the structure contains the eutectic Co-C-Al-W (white irregular formations) with fine precipitated particles of the same composition as that of solid solution B. There are also regular and irregular dark, sharp-edged particles in the structure, which reach a size in the range of 20–60 µm and have a high tungsten content. Fine dark fibrous formations are visible in the structure of these particles (Figure 2b).

**Figure 3:**
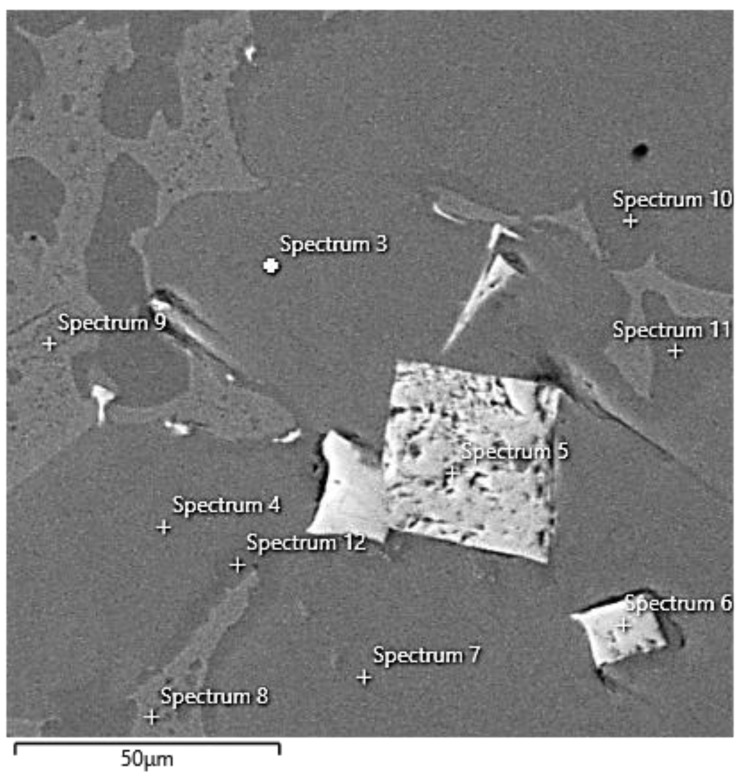
SEM image of Co70W12Al15 alloy with EDS point analysis (Spectra 3–12).

A TESCAN VEGA 3 XMU scanning electron microscope with an Oxford EDS analyzer was used for a more detailed study of the microstructure. Solid solution A, solid solution B, eutectic, and sharp-edged light particles were examined by point analysis (Figure 3, Table 2). All compositions are in wt.%. Point analyses of solid solution A (spectrum 3, 4, 7, 12) show that the basis is cobalt 64.55 ± 0.31%; then carbon 18.60 ± 0.32% and aluminum 10.80 ± 0.54%; and smaller amounts of tungsten 3.10 ± 0.22%, silicon 1.40 ± 0.22%, and iron 1.05 ± 0.06%. Point analyses of solid solution B (spectrum 10, 11) show that the base is cobalt 68.10 ± 0.14%; then carbon 19.95 ± 0.07% and aluminum 8.8%; as well as smaller amounts of tungsten 1.7 ± 0.14%, silicon 0.3%, and iron 0.9%. Comparing the point EDS analyses for solid solutions A and B, it can be stated that the basic difference between the two solid solutions is in the cobalt content, where the lighter solid solution A contains about 4–5% less cobalt, but on the other hand contains a little more aluminum, tungsten, and silicon. The eutectic (spectrum 8, 9) contains approximately 69.10 ± 0.14% cobalt, 18.65 ± 0.07% carbon, 5.50 ± 1.70% aluminum, 3.95 ± 0.49% tungsten, 0.85 ± 0.49% silicon, and 1.15 ± 0.21% iron. Point analyses of sharp-edged particles (spectrum 5, 6) show that the basis is tungsten 45.80 ± 3.68%, titanium 27.70 ± 4.67%, carbon 24.35 ± 0.78%, and cobalt 1.95 ± 1.63%. Thus, they are probably irregular metal carbide particles—in the case of this alloy, a complex carbide of tungsten and titanium ((W, Ti) C), which was also confirmed by X-ray diffraction.

### 3.3. Mechanical Properties of the Produced Alloy Co70W12Al15

The mechanical properties of the individual phases were investigated using a microhardness tester according to the Vickers test with a load of 100 g (HV 0.1). The results are shown in Table 3. The lowest hardness was shown by the eutectic structure, at 439.0 ± 34.5 HV 0.1. Both solid solutions differ slightly in hardness; solid solution A has a higher hardness (669.0 ± 80.5 HV 0.1), while solid solution B reaches a hardness of 524.4 ± 88.4 HV 0.1. Sharp-edged particles reach a very high hardness; the average hardness is 3252.7 + 356.2 HV 0.1.

## 4. Discussion

For the preparation of a special multicomponent alloy Co70W12Al15 by the aluminothermic reaction, aluminum, tungsten oxide, cobalt dioxide, pulverized coal, and a slag-forming additive—calcium fluoride—were used. Calcium fluoride has a lower density (3.180 g⋅cm^−3^ [54]) compared to the produced alloy and will be on the surface of the melt after the reaction. This separates the slag in the upper part of the crucible and the liquid metal as a complex alloy, which is heavier and sinks to the bottom of the crucible. The aim of the slag-forming additive is to protect the melt from the surrounding atmosphere and to remove metal oxides and other impurities from the alloy into the slag. The mixture was heated to approximately 1000 °C to initiate the aluminothermic reaction. During the reaction, metal oxides were reduced with aluminum (entering the slag) and the metals (especially Co, W, Ti) were partially reacted with carbon to form carbides of the respective metals.

After the reaction, the slag was separated and the remaining alloy was crushed after solidification and then ground in a planetary mill to obtain a fine powder for coating.

### 4.1. Comparison of Composition of Prepared Co70W12Al15 by XRF and EDS Analysis

Table 4 compares the chemical composition of the sample obtained by XRF and area EDS analysis. The results in the table show there is not much difference between X-ray fluorescence (XRF) and energy dispersive spectroscopy (EDS). Using EDS analysis, it is also possible to determine the carbon and oxygen content, which cannot be determined using XRF.

Table 5 characterizes the individual phases in terms of their chemical composition and mechanical properties (microhardness). The highest hardness is achieved by sharp-edged particles, which, depending on their composition, correspond to tungsten carbide or titanium tungsten carbide. On the contrary, the particles with the lowest hardness have a eutectic structure; at the same time, they contain the most cobalt and the least aluminum. Solid solutions A and B differ by about 150 HV 0.1, while harder solid solution A contains less cobalt and carbon but more aluminum, tungsten, and silicon.

### 4.2. Comparison of Prepared Co70W12Al15 Alloy with Other Cobalt Alloys

The produced alloy Co70W12Al15 contains an admixture of titanium, iron, and calcium. It can be considered a cobalt alloy with a specific composition. Table 6 shows the composition of known cobalt alloys—namely, wear-resistant alloys, high-temperature alloys, or corrosion-resistant alloys. Corrosion-resistant cobalt alloys, in terms of corrosion resistance, can be compared with corrosion-resistant steel and related to the formation of a passive Cr_2_O_3_ layer. In addition, the alloying of molybdenum into these alloys refines their structure, and this significantly increases the strength properties of these alloys in both cast and wrought states [55].

Another separate group is biomedical cobalt alloys. For this use of cobalt alloys, we have the following types of cobalt alloys: ASTM F75—Co-28Cr-6Mo (foundry alloy), ASTM F90—Co-20Cr-15W-10Ni (alloy for forming), and AST F62—Co-35Ni-20Cr-10Mo (alloy for forming). The chemical composition of these alloys is given in Table 7 [55,56].

The alloy Co70W12Al15 produced in the presence of titanium, iron, carbon, and calcium cannot be included with any of the commercially produced cobalt alloys. The reason for this is the high content of aluminum in the Co70W12Al15 alloy, as well as the high content of titanium, calcium, and carbon. On the other hand, the Co70W12Al15 alloy produced is practically free of chromium, molybdenum, and nickel [57].

## 5. Conclusions

A new alloy Co70W12Al15 was successfully prepared by aluminothermic reaction. This alloy was used for the subsequent preparation of a special composite nanopowder and for the surface coating aluminum, magnesium, and iron alloys. The results obtained can be summarized in the points below.

(1) The results of XRF analysis (chemical composition of the alloy) showed that the alloy consists of 68% cobalt; 15% aluminum; 13% tungsten; and small amounts of calcium, titanium, and iron from the aluminothermic reaction.
(2) In addition to the results of the XRF analysis, the chemical composition of the EDS analysis also showed the presence of carbon and oxygen.
(3) The phase composition determined by X-ray diffraction was characterized by the AlCo phase, tungsten carbide, titanium tungsten carbide, silica, and carbon.
(4) Using laser and scanning electron microscopy, the samples were characterized in terms of their microstructure. The microstructure was composed of two solid solutions, which contained mainly cobalt, carbon, and aluminum; a eutectic, which has a lower aluminum content than both solid solutions; and sharp-edged particles of tungsten carbide titanium tungsten and carbide, which, according to EDS analysis, correspond to XRD analysis.
(5) The microhardness results showed a difference between the two solid solutions (solid solution A was 150 HV 0.1 harder than solid solution B), the eutectic (which had the lowest hardness), and the carbide particles (which were very hard (more than 3000 HV 0.1)).

An important result of the research is the revelation of the possibility to, through the aluminothermic reaction of refractory metals (W, Co, Ti), convert a large part of these metals into their corresponding carbides.

## Figures and Tables

**Table 1:** Results of the chemical composition of the produced Co70W12Al15 alloy analyzed by an X-ray fluorescence spectrometer.

Chemical Element [wt.%]	Measurement 1	Measurement 2	Measurement 3	Average	Standard Deviation
Al	14.90	15.10	15.44	15.15	0.27
Ca	1.67	3.48	2.99	2.71	0.94
Ti	2.26	1.31	2.66	2.08	0.69
Cr	0.27	0.24	0.25	0.25	0.02
Mg	0.14	0.17	0.18	0.16	0.02
Fe	2.34	2.67	3.10	2.70	0.38
Co	66.09	68.78	69.31	68.06	1.73
Cu	0.16	0.18	0.13	0.16	0.03
Mo	0.30	0.25	0.34	0.30	0.05
W	11.88	11.97	14.00	12.62	1.20

**Table 2:** EDS point analysis of Co70W12Al15 alloy.

Chemical Element [wt.%]	Spectrum 3	Spectrum 4	Spectrum 5	Spectrum 6	Spectrum 7	Spectrum 8	Spectrum 9	Spectrum 10	Spectrum 11	Spectrum 12
Co	64.8	64.6	0.8	3.1	64.7	69.0	69.2	68.0	68.2	64.1
C	18.8	18.5	24.9	23.8	18.2	18.7	18.6	19.9	20.0	18.9
Al	10.5	10.6	0	0	11.6	6.7	4.3	8.8	8.8	10.5
W	3.0	3.1	43.2	48.4	2.9	3.6	4.3	1.8	1.6	3.4
Si	1.4	1.5	0	0	1.1	0.5	1.2	0.3	0.3	1.6
Fe	1.1	1.1	0	0	1.0	1.0	1.3	0.9	0.9	1.0
Ti	0	0	31.0	24.4	0	0	0	0	0	0

**Table 3:** Microhardness (HV 0.1) of Co70W12Al15 alloy.

Measurement	Solid Solution A	Solid Solution B	Eutectic Structure	Particles
1.	701	634	448	3301
2.	613	418	493	2956
3.	574	603	398	3428
4.	781	424	421	3433
5.	766	549	493	3185
6.	613	460	429	2758
7.	678	402	447	3422
8.	613	594	391	4020
9.	584	585	447	3064
10.	767	575	423	2960
Average	669.0	524.4	439.0	3252.7
Standard deviation	80.5	88.4	34.5	356.2

**Table 4:** Results of the chemical composition of the produced Co70W12Al15 alloy analyzed by X-ray fluorescence spectrometer (XRF) and area EDS analysis.

Chemical Element [wt.%]	Average (XRF)	Average (EDS)
Al	15.15	15.53
Ca	2.71	-
Ti	2.08	1.11
Cr	0.25	-
Mg	0.16	-
Fe	2.70	-
Co	68.06	63.49
Cu	0.16	-
Mo	0.30	-
W	12.62	12.00
C	-	6.07
O	-	1.80

**Table 5:** Results of the phase composition of the produced Co70W12Al15 alloy analyzed by X-ray fluorescence diffractometer (XRD) and microhardness HV 0.1.

Phase	Average Composition (EDS, wt.%)		HV 0.1
Solid solution A	Co	64.55	669.0
	C	18.60	
	Al	10.80	
	W	3.10	
	Si	1.40	
	Fe	1.05	
Solid solution B	Co	68.10	524.4
	C	19.95	
	Al	8.80	
	W	1.70	
	Si	0.30	
	Fe	0.90	
Eutectic	Co	69.10	439.0
	C	18.65	
	Al	5.50	
	W	3.95	
	Si	0.85	
	Fe	1.15	
Particles	W	45.80	3252.7
	Ti	27.70	
	C	24.35	
	Co	1.95	

**Table 6:** Chemical composition of special cobalt alloys.

Chemical Element [wt.%]	Wear-Resistant Alloys (Stellite Alloys)	Alloys for High Temperatures	Stainless Alloys
Cr	25–30	20–33	20–25
Mo	<1	-	5–10
W	2–15	7–15	<2
C	0.25–3.3	0.1–0.6	<0.8
Fe	<3	<3	<3
Ni	<2	-	9–35
Si	<2	-	-
Mn	<1	-	-
Co	remain	remain	remain

**Table 7:** Chemical composition of biomedical cobalt alloys.

Chemical Element [wt.%]	F75	F90	F62
Cr	27–30	19–21	19–21
Mo	5–7	-	9–10.5
W	<0.2	14–16	-
C	<0.35	<0.15	<0.025
Fe	<0.75	<3	<1
Ni	<2.5	9–11	33–37
Si	<1	<0.4	<0.15
Mn	<1	<3	<1
Co	58.9–69.5	45.5–56.2	29.0–38.8

## Data Availability

Data are contained within the article.
